# Genetics and Physiology of the Nuclearly Inherited Yellow Foliar Mutants in Soybean

**DOI:** 10.3389/fpls.2018.00471

**Published:** 2018-04-11

**Authors:** Devinder Sandhu, Zachary Coleman, Taylor Atkinson, Krishan M. Rai, Venugopal Mendu

**Affiliations:** ^1^USDA-ARS Salinity Laboratory, Riverside, CA, United States; ^2^Department of Biology, University of Wisconsin-Stevens Point, Stevens Point, WI, United States; ^3^Fiber and Biopolymer Research Institute, Department of Plant and Soil Science, Texas Tech University, Lubbock, TX, United States

**Keywords:** chlorophyll deficient, chloroplast, photosynthesis, photosynthetic pigments, soybean, yellow mutant

## Abstract

Plant photosynthetic pigments are important in harvesting the light energy and transfer of energy during photosynthesis. There are several yellow foliar mutants discovered in soybean and chromosomal locations for about half of them have been deduced. Viable-yellow mutants are capable of surviving with decreased photosynthesis, while lethal-yellow mutants die shortly after germination. In addition to the decreased chlorophyll content, other features associated with yellow mutants include altered Chl *a* and Chl *b* ratio, reduction in chloroplast size and number, lower levels of other photosynthetic pigments, inability of thylakoids to stack into granum, lack of lamellae to interconnect granum and reduced size of the light harvesting complex. For some yellow mutants, temperature and/or light play a critical role in the manifestation of phenotype. Although yellow foliar mutants are viewed as undesirable for crop production, there is the possibility of these mutants to create a positive impact by reducing the total amount of chlorophyll and diverting resources toward increased biochemical photosynthetic capacity leading to increased yield. Recent advances in model plants led to the isolation and characterization of various genes associated with yellow foliar phenotype. Knowledge gained from the model plants can be applied using homology based cloning approach to isolate genes in soybean and understanding the modes of actions of the involved proteins. Identifying and characterizing yellow foliar mutants will not only aid in understanding the biosynthetic pathways involved in the photosynthetic machinery, but may also provide ways to increase soybean productivity.

## Introduction

Soybean is among the world’s most valuable food and feed crops with high levels of protein (∼40%) and oil (∼20%) content, which makes it important for human nutrition, livestock, and aquaculture feed (Singh and Hymowitz, 1999; Masuda and Goldsmith, 2009). In the United States, soybean is the second most planted field crop behind corn with estimated cultivation area of 89.5 million acres in 2017^1^. Soybean has become an increasingly important staple food and is consumed every day in a variety of forms. Simultaneously, soybean crop has also gained tremendous share in other commercial applications such as biodiesel, candles, biocomposites, adhesives, environmentally friendly solvents to remove oil from water, crayons, lubricants, hydraulic fluid, and many other uses.

Photosynthesis is an essential process that helps the plant to harvest the sunlight, or light energy, and convert it into chemical energy (Jagannathan and Golbeck, 2009). Light is absorbed by photosystems and excites the electrons present within the pigments to a higher energy level. The excited electrons are taken up by primary electron acceptors and are passed on through an electron transport chain located within the thylakoid membranes (Jagannathan and Golbeck, 2009). While the charged electrons travel through the transport chain, the energy reduction that occurs is captured and employed to push protons (H^+^) through the membrane from the stroma to the lumen. The transport of protons back from the lumen to the stroma creates a proton-motive force which produces adenosine triphosphate (ATP), the energy currency of the cell (Jagannathan and Golbeck, 2009). This entire process occurs within the chloroplast, a semi-autonomous organelle which evolved approximately 1–1.5 billion years ago due to a cyanobacterial ancestor engulfment event by a eukaryote cell (Reyes-Prieto et al., 2007). The majority of the ancestral bacterial genes was either lost in due course of evolutionary process or transferred to the nuclear genome of the host (Sakamoto et al., 2008). Hence, several important components of chloroplast are generated by the nucleus-encoded pre-proteins (Jarvis et al., 1998; Bauer et al., 2000; Chen et al., 2002; Chou et al., 2003; Inaba et al., 2003; Kubis et al., 2003; Ivanova et al., 2004). In fact, approximately 80–90% of the chloroplast proteins are encoded by nuclear genes (Jarvis et al., 1998; Jarvis, 2001). In order for the chloroplast to be produced properly ∼3,000 different proteins must be encoded from the nuclear DNA and imported to the chloroplast (Inaba et al., 2003; Ivanova et al., 2004). These precursor proteins are synthesized on ribosomes in the cytosol (Kubis et al., 2003). A transit peptide present in each of these proteins acts as a signal which allows the pre-protein to be imported into the chloroplast and pass through the chloroplast membrane (Bauer et al., 2000; Chen et al., 2002; Chou et al., 2003; Inaba et al., 2003; Kubis et al., 2003; Ivanova et al., 2004). The transit peptide is recognized and associates with its specific Toc (translocon at the outer envelope membrane of chloroplasts) complex which facilitates the translocation of the pre-protein into the chloroplast (Chou et al., 2003; Inaba et al., 2003; Kubis et al., 2003; Ivanova et al., 2004). As the pre-protein travels through the outer membrane, the transit peptide is then recognized by a specific Tic (translocon at the inner envelope membrane of chloroplast) complex (Chou et al., 2003; Inaba et al., 2003; Kubis et al., 2003; Ivanova et al., 2004). Once the peptide associates with the proper Tic complex, it can then pass through the inner membrane into the stroma. When the pre-protein moves into the stroma the transit peptide is removed (Chou et al., 2003) and it is folded into its final confirmation with the assistance of molecular chaperones (Kubis et al., 2003). The Toc/Tic complexes are extremely important for the translocation of essential proteins to make the chloroplast a functional photosynthetic organelle. Photosystems I and II, which are vital for photosynthesis, are housed within the thylakoid membranes (Jagannathan and Golbeck, 2009). These thylakoids contain pigments that play roles in harvesting light and transferring energy in reaction centers during photosynthesis (Espinosa, 2014).

**FIGURE 1:**
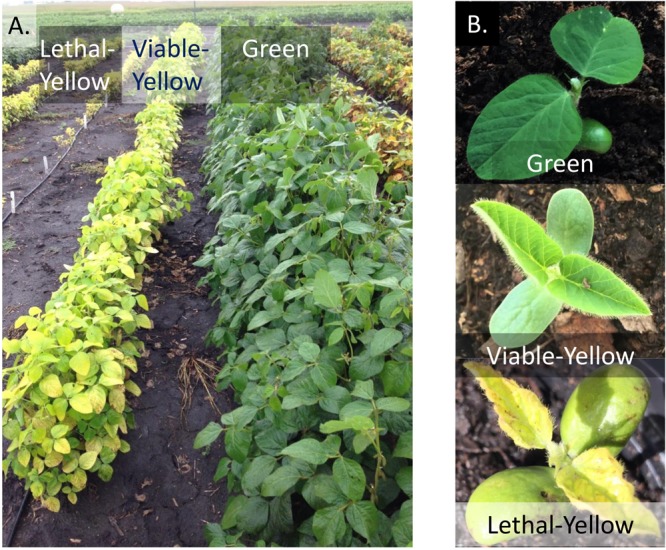
Physical appearances of wild-type green line in comparison to a lethal-yellow and a viable-yellow mutant. **(A)** Plants grown in field conditions. **(B)** Plants grown in greenhouse conditions.

Photosynthesis is a complex and tightly regulated process which involves several enzymes and biochemical reactions (Johnson, 2016). If a gene that encodes for an enzyme involved in biosynthesis of a pigment or in a metabolic step is mutated, it results in reduced photosynthesis. These mutants are designated as yellow mutants or chlorophyll-deficient mutants (Emerson, 1929) (**Figure 1**). In addition to the yellowing of the leaves many of the mutations cause secondary problems that can affect numerous other functions within the plant. Here, we have focused on nuclearly inherited yellow foliar mutants in soybean. Several nuclearly inherited yellow foliar mutants have been identified and characterized in soybean and most of them are governed by a single recessive gene (**Table 1**). In spite of their widespread occurrence, relatively few yellow foliar mutants have been identified and characterized at the molecular level in soybean. For the characterization of remaining mutants, some questions of utmost interest are: (1) What is the gene and its product? (2) What biological mechanism is it involved in? (3) How does it regulate the yellow foliar phenotype?

**Table 1:** Yellow foliar mutants in soybean.

S. No.	Mutant/gene name	Strain	Viable or lethal	Chromosomal location	Phenotypes	Sources
1	*y1*	–	Viable	Unknown	Greenish-yellow, weak plant	Morse and Cartter, 1937
2	*y3*	T139	Lethal	Unknown	Leaves turn yellow as plant grows. Variable chlorophyll content during life cycle, reduced photosynthetic pigments, reduced leaf area, early senescence and early death.	Morse and Cartter, 1937; Eskins and Banks, 1979; Eskins et al., 1983; Jiang et al., 1997
3	*y4*	T102	Viable	Unknown	Greenish yellow leaves, weak plant	Morse and Cartter, 1937; Woodworth and Williams, 1938
4	*y5*	T116, T134	Viable	Unknown	Greenish yellow leaves	Morse and Cartter, 1937; Woodworth and Williams, 1938
5	*y6*	T136	Viable	Unknown	Pale green leaves in early plant stages. Later turns to normal green.	Morse and Cartter, 1937; Woodworth and Williams, 1938
6	*y7*	T138, T144	Viable	Unknown	Yellowing starts from stem, then leaves, pods, and seeds. In leaves, it appears in veins first. Yellow growth in cool weather.	Morse and Cartter, 1937; Woodworth and Williams, 1938; Eskins and Banks, 1979
7	*y8*	T144	Viable	Unknown	Yellow green leaves at younger stages. Later plant is undistinguishable from normal green. Yellow growth in cool weather.	Probst, 1950; Eskins and Banks, 1979
8	*y9*	T135	Viable	15	Yellow at seedling stage. Greenish-yellow by maturity.	Probst, 1950; Eskins and Banks, 1979; Eskins et al., 1983; Ghirardi and Melis, 1988; Thorson et al., 1989; Devine, 1998; Zou et al., 2003; Palmer and Xu, 2008
9	*y10*	T161	Viable	3	Greenish-yellow seedling	Probst, 1950; Zou et al., 2003
10	*y11*	T219	Lethal	13	Bright greenish-yellow leaves as heterozygote and yellow lethal as homozygote.	Weber and Weiss, 1959; Sun, 1963; Wolf, 1963; Keck and Dilley, 1970; Keck et al., 1970; Crang and Noble, 1974; Palmer et al., 1979; Eskins et al., 1983; Ghirardi and Melis, 1988; Campbell et al., 2015
11	*y12*	T233	Viable	6	Whitish primary leaves, yellowish green mature leaves	Weiss, 1970a
12	*y13*	T230	Viable	13	Whitish-green seedlings, greenish-yellow leaves	Weiss, 1970b
13	*y14*	T229	Viable	Unknown	Light green leaves	Nissly et al., 1976
14	*y16*	T257	Lethal	Unknown	Nearly white. Flower buds were formed but no seeds were recovered. Plants died early.	Wilcox and Probst, 1969
15	*y17*	T162	Viable	15	Light yellowish green leaves	Nissly et al., 1981; Devine, 1998; Palmer and Xu, 2008
16	*y18/ y18_1*	T218H, T225H	Lethal	14	Yellow leaves	Cheng, 1993; Burzlaff and Palmer, 1999; Kato and Palmer, 2004
17	*y18_2*	T362H	Lethal	17	Yellow leaves	Kato and Palmer, 2004
18	*y18_m*	T218M	Viable	Unknown	Unstable allele resulting in chlorophyll chimera	Peterson and Weber, 1969; Cheng, 1993; Kato and Palmer, 2004
19	*y19*	T265H	Lethal	Unknown	Delayed albino	Palmer et al., 1990
20	*y20*	T234, T253, T317, T323-325, T334-351, T361	Viable	12	Yellowish-green leaves, weak plant	Palmer, 1984; Burzlaff and Palmer, 1999; Chen et al., 1999; Espinosa, 2014; Sandhu et al., 2017
21	*y21*	Shennong 2015	Lethal	Unknown	Yellow leaves	Yu et al., 1986
22	*y22*	T270H	Conditional lethal. Viable in greenhouse, lethal in field	Unknown	Greenish yellow leaves, very weak plant	Stelly et al., 1979; Palmer et al., 1990
23	*y23*	T288	Viable	13	Leaves turn green to yellow-white and turn necrotic. Wilting and defoliation after germination. Weak plant	Palmer et al., 1990
24	*yl_PR350*	–	Lethal	15	Yellow leaves	Reed et al., 2014
25	*psbP*	T378H	Lethal	3	Yellow leaves. Plant dies within 2–3 weeks	Sandhu et al., 2016
26	*tic110*	T379, T380, T381	Viable	2	Light green leaves	Sandhu et al., 2016
27	*cd1*	–	Viable	10	Yellowish-green leaves	Zhang et al., 2011
28	*CD-5*	–	Lethal	15	Yellowish-green leaves, short plant	Palmer et al., 1989; Campbell et al., 2015

In the last few decades tremendous research has been conducted on understanding metabolic pathways in model genetic organisms. For instance, a large number of yellow foliar mutant genes have been isolated and characterized in rice and Arabidopsis, and their roles in biological mechanisms were determined. With the advancement of genomic and genetic approaches and availability of mounting body of knowledge, it is now feasible to extend genetic and biological information from model species to crop plants. In this report, we have discussed the roles of different proteins characterized in model plants that are involved in yellow foliar phenotypes and how that information can become instrumental in characterizing additional mutants in soybean.

### Identification, Inheritance, and Characterization of Yellow Foliar Mutants

The discovery of the first yellow foliar mutation in soybean was not well-documented; however, by 1940 there were a few yellow foliar mutants known, which were described as *yellow 1* (*y1*), *y2*, and so on (Morse and Cartter, 1937; Probst, 1950; Johnson and Bernard, 1962). Since then, many additional yellow foliar mutants have been identified in soybean.

Chlorophyll deficient mutants are broadly classified into two groups, viable-yellows and lethal-yellows. Viable-yellow mutant plants are capable of surviving with pale green or yellow/green leaves (Palmer et al., 2000; Sandhu et al., 2016) (**Figure 1**). As the seedlings grow, the plant may either outgrow the yellow leaves or retain the phenotype for the duration of its life depending on the mutated gene. Lethal-yellow mutant seedlings are initially yellow colored and have stunted growth, but are incapable of surviving. The plants vary in their length of survival from only for a few days after germination to a few weeks (Palmer et al., 2000; Sandhu et al., 2016).

Several mutants including *y3, y11, y16, y18/y18_1, y18_2, y19, y21, yl_PR350, psbP*, and *CD-5* are considered lethal-yellows due to the early death of the plants (**Table 1**). One of the chlorophyll deficient mutant, *y22*, is considered conditional lethal as it produces seeds in a greenhouse; however, mutant plants die shortly after flowering in the field (Palmer et al., 1990). *Y18-m* was identified as an unstable mutable allele in soybean variety “Lincoln” that showed variegated yellow sectors of variable sizes (Peterson and Weber, 1969). *Y18-m* can change to stable forms *Y18* or *y18*, or may remain unstable (Peterson and Weber, 1969) (**Table 1**). Inheritance study of two lethal-yellow mutants {T218H (*y18*) and T225H (*y18_1*)} from the progeny of *Y18-m* revealed that *y18* and *y18_1* were allelic (Kato and Palmer, 2004) (**Table 1**). The *CD-5* and *y11* mutants that exhibited similar phenotypes had identical amino acid substitution in paralogous genes (Campbell et al., 2015). *Y11, Y18/Y18_1, Y18_2, YL_PR350, PsbP*, and *CD-5* were genetically mapped to chromosomes 13, 14, 17, 15, 3 and 15, respectively (Mahama et al., 2002; Kato and Palmer, 2004; Reed et al., 2014; Campbell et al., 2015; Sandhu et al., 2016).

The remaining 18 yellow mutants (*y1, y4, y5, y6, y7, y8, y9, y10, y12, y13, y14, y17, y18-m, y20, y22, y23, tic110*, and *cd1*) are viable (**Table 1**). Some mutants such as *y6, y8, y10*, and *y13* develop yellow–green leaves in young plants but later stages develop more and more chlorophyll and become visually indistinguishable from normal green plants (Woodworth and Williams, 1938; Probst, 1950; Weiss, 1970b). On the contrary, the *y23* mutant represents a weak plant which changes from green to yellow-white and slowly turns necrotic (Palmer et al., 1990). *Y9, Y10, Y12, Y13, Y17, Y20, Y23, tic110*, and *Cd1* were genetically mapped to chromosomes 15, 3, 6, 13, 15, 12, 13, 2, and 10, respectively (**Table 1**).

Of 28 unique yellow foliar mutants known in soybean, 15 have been mapped to soybean chromosomes (**Table 1**). The genetic locations for the remaining 13 mutants are unknown.

### Variations in the Photosynthetic Pigments

**FIGURE 2:**
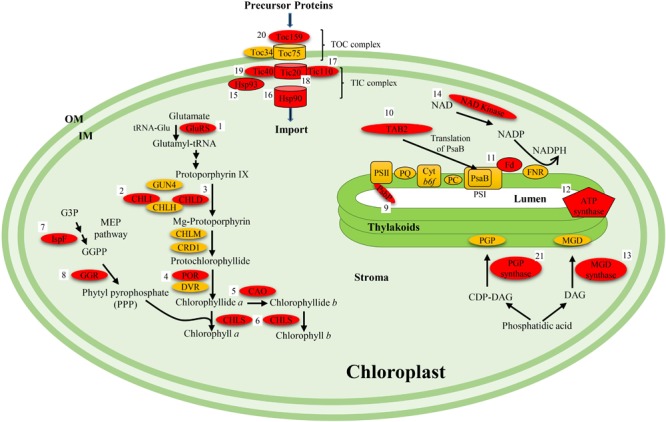
Summary of various proteins involved in yellow foliar phenotypes in plants showing their metabolic functions. The characterized proteins that have been functionally linked with the yellow phenotype are highlighted in red. Numbers 1 through 21 represent names of the mutants identified for the proteins represented in the pathway. (1) *cde1* (*Os*); (2) *chl9* (*Os*), *y11* (*Gm*), and *cd5* (*Gm*); (3) *ygl7* (*Os*), *ygl7* (*Os*), *chl1* (*Os*), and *ygl98* (*Os*); (4) *fgl* (*Os*); (5) *cao1* (*Os*); (6) *ygl1* (*Os*); (7) *505ys* (*Os*); (8) *lyl1-1* (*Os*); (9) *psbP* (*Gm*); (10) *tab2* (*Gm*); (11) *501ys* (*Os*); (12) *yl1* (*Os*) and *ys83* (*Os*); (13) *mgd1* (*At*); (14) *nadk2* (*At*); (15) *hsp93* (*At*); (16) *hsp90c* (*At*); (17) *tic110* (*Gm*); (18) *tic20* (*At*); (19) *tic40* (*At*); (20) *toc159* (*At*); (21) *pgp1* (*At*). Abbreviations in the parenthesis after the mutant name represent the species in which the mutant was identified (*At, Arabidopsis thaliana*; *Os, Oryza sativa*; *Gm, Glycine max*). GluRS, glutamyl-tRNA synthase; GUN4, genomes uncoupled 4; CHLD, CHLH, CHLI are subunits of the Mg-chelatase enzyme; CRD1, copper response defect 1; POR, protochlorophyllide oxidoreductase; DVR, divinyl reductase; CAO, chlorophyll *a* oxygenase; CHLS, chlorophyll synthase; TICs, translocon at the inner envelope membrane of chloroplast; TOCs, translocon at the outer envelope membrane of chloroplast; PSI, photosystem I; PSII, photosystem II; PQ, plastoquinone; PC, plastocyanin; Cyt *b_6_F*, cytochrome *b6f*; PsbP, component protein of PSII; DAG, diacylglycerol; CDP-DAG, cytidine diphosphate diacylglycerol; PGP, phosphatidyl glycerol phosphate; MGD, monogalactosyl diacylglycerol; TAB2, translation of PsaB 2; PsaB, component protein of PSI; Hsp, heat shock protein; NAD, nicotinamide adenine dinucleotide; FNR, ferredoxin NADP^+^ reductase; G3P, glycerlaldehyde 3-phosphate; GGPP, geranylgeranyl pyrophosphate; OM, outer membrane; IM, inner membrane.

A common characteristic of the yellow foliar mutants is a decrease in the total amount of chlorophyll which subsequently reduces photosynthetic activity. Chlorophyll synthesis is a multistep process that requires several enzymes (Johnson, 2016). Chlorophyllide esterification that is catalyzed by chlorophyll synthase is the last step in the chlorophyll biosynthesis process in plants. A missense mutation in the *chlorophyll synthase* gene that affected its enzyme activity resulted in *yellow green leaf1* (*ygl1*) mutant in rice (Wu et al., 2007) (**Figure 2**). Protochlorophyllide oxidoreductase B (PORB) that is constitutively expressed throughout the leaf development is required for light dependent chlorophyll synthesis (Sakuraba et al., 2013). The *faded green leaf* (*FGL*) gene in rice encodes OsPORB, which catalyzes protochlorophyllide to chlorophyllide in chlorophyll synthesis (Sakuraba et al., 2013) (**Figure 2**). In the *fgl* mutant, the excessive accumulation of reactive oxygen species due to increased levels of non-photoactive protochlorophyllide led to the downregulation of the chlorophyll synthesis or photosynthesis related genes, resulting in the variegated leaf phenotype (Sakuraba et al., 2013). Magnesium chelatase that catalyzes the insertion of Mg^2+^ into the center of protoporphyrin IX contains three subunits (ChlH, ChlD, and ChlI) and is a key enzyme in chlorophyll biosynthesis (Johnson, 2016) (**Figure 2**). In rice, the yellow green leaf phenotype of the *ygl7* mutant was due to a missense mutation in the gene encoding for magnesium-chelatase ChlD protein (Deng et al., 2014). Although, three additional yellow green rice mutants, *chlorina-1, ygl98*, and *ygl3* display slightly different phenotypes, they represent different alleles of the *ChlD* gene (Deng et al., 2014) (**Figure 2**). Another rice mutant, *chlorina-9*, was shown to be the result of mutation in the gene encoding for ChlI subunit (Zhang et al., 2006). Similarly, two chlorophyll deficient phenotypes in soybean, *y11* and *CD-5*, were due to mutations in paralogous genes encoding ChlI1a and ChlI1b (Campbell et al., 2015) (**Figure 2**).

In soybean, the majority of mutants cause a 30–66% reduction in overall chlorophyll content (Shoemaker et al., 1985; Zhang et al., 2011; Sandhu et al., 2016). Lethal-yellow mutants have considerably higher reduction in chlorophyll content as compared to viable-yellow mutants. For instance, chlorophyll content was reduced by ∼92% in the *psbP* (T378H) mutant (Sandhu et al., 2016). Nevertheless, the chlorophyll content of the mutants may not remain constant throughout the life cycle. Different mutants display diverse chlorophyll profiles during their development. For example, *y3* showed higher chlorophyll content as compared to *y11* at 35 days after planting; however, *y3* showed less chlorophyll than *y11* at 50 days after planting (Jiang et al., 1997).

In addition to the overall amount of chlorophyll, mutations also have an impact on the chlorophyll *a/b* ratio. Within each leaf there is a balance between the two different kinds of chlorophylls: chlorophyll *a* and chlorophyll *b*. Chlorophyll *a* is the most abundant pigment that absorbs wavelengths in the blue and red color range and Chlorophyll *b* primarily absorbs blue light. A mutation in *OsCAO1* gene that encodes a chlorophyll *a* oxygenase, the enzyme responsible for the catalysis of chlorophyll *a* into chlorophyll *b*, was responsible for the pale green phenotype in rice (Lee et al., 2005) (**Figure 2**). In the majority of soybean mutants both types of chlorophylls have been reduced (Eskins and Banks, 1979; Eskins et al., 1983; Sandhu et al., 2016). However, when comparing the wild-type green and the yellow foliar mutants, the ratio of chlorophyll *a/b* was increased due to a more drastic decrease in chlorophyll *b* content then chlorophyll *a* (Shoemaker et al., 1985; Jiang et al., 1997; Zhang et al., 2011). Specifically, the *y3, y7, y8, y9, y11*, and *cd1* mutants exhibited increased chlorophyll *a/b* ratio as compared to wild-type plant during leaf ontogeny (Eskins and Banks, 1979; Palmer et al., 1979; Eskins et al., 1983; Shoemaker et al., 1985; Ghirardi and Melis, 1988; Jiang et al., 1997; Zhang et al., 2011). Although, the majority of chlorophyll mutants displays an increased chlorophyll *a/b* ratio, however, some yellow foliar mutants (e.g., *y18-m, psbP*, and *tic110*) demonstrate no change in the chlorophyll *a/b* ratio (Cheng and Chandlee, 1999; Sandhu et al., 2016).

In addition to the synthesis, accumulation of chlorophyll is also very important in plants. The *YLC1* (*young leaf chlorosis 1*) gene encodes DUF3353 family protein in rice that is targeted to chloroplast and is essential for chlorophyll and lutein accumulation during early leaf development (Zhou K. et al., 2013).

Chlorophylls are not the only photosynthetic pigments used by plants for photosynthesis; in fact there are typically several other pigments involved in photosynthetic process. Along with chlorophyll *a* and *b* there is lutein, carotene, violaxanthin, and neoxanthin (Eskins and Banks, 1979; Eskins et al., 1983). At least five soybean mutants (*y3, y7, y8, y9*, and *y11)* are known to directly impact the content of the other four photosynthetic pigments (Eskins and Banks, 1979; Eskins et al., 1983). All five mutants displayed decrease in the total amount of each pigment present in the leaves. Neoxanthin was drastically reduced while carotene, lutein, and violaxanthin were slightly reduced. Furthermore, the ratios of chlorophyll *a* to each pigment in the mutants, were distinctly different from the normal green soybean plant (Eskins et al., 1983). The ratio of chlorophyll *a* to neoxanthin amplifies significantly, whereas chlorophyll *a* to carotene, lutein, or violaxanthin ratios decrease.

The antennae are light harvesting complexes (LHCs) in chloroplast and are required for both photosystems to function properly. They are typically embedded into the thylakoid membranes. The LHCs absorb light energy and funnel it to the reaction center during photosynthesis. Certain mutations can actually decrease the size of the antennae which would affect the light harvesting capabilities of both photosystems. The *y9* soybean mutant gene is a primary example of how photosynthesis is affected by reducing the amount of the LHC II polypeptides (Ghirardi and Melis, 1988). Overall the efficiency to undergo photosynthesis is decreased due to the reduction in available LHC II and the inability of LHC II to bind. At the same time the ratio between photosystem II (PSII) to photosystem I (PSI) have also been affected (Ghirardi and Melis, 1988). The mutants *y3, y9*, and *y11* displayed increased relative ratio of PSII to PSI (Eskins et al., 1983). Due to this drastic increase, photosynthesis cannot function properly because there are not enough PSIs to take in the charged electrons from all of the PSIIs. Defects in biogenesis or assembly of photosystems often result in yellow mutants. For instance, for the *YL_PR350* lethal-yellow mutant, a putative candidate gene was identified that codes for TAB2 protein known to play a role in biogenesis of photosystems I and II (Reed et al., 2014). Another lethal-yellow soybean mutant (T378H) was found to have a single base insertion in *GmPsbP*, an extrinsic protein of PSII which is critical for oxygen evolution during photosynthesis (Sandhu et al., 2016) (**Figure 2**). Reduction in photochemical conversion efficiency in *psbP* soybean mutant suggested damage to PSII (Sandhu et al., 2016). A stay-green mutant, in which chlorophyll degradation is impaired, is controlled by maternally inherited *cytG* gene that encodes the PsbM protein, an important component of the small subunits of PSII (Kohzuma et al., 2017). Interestingly, the *y3* mutation that is nuclearly inherited is known to suppress the cytG phenotype (Kohzuma et al., 2017). The isolation and characterization of *Y3* may shed light on underlying mechanism and the type of interaction between the Y3 and PsbM proteins.

### Defects in Chloroplast Development

The chloroplast is one of the most important organelles present in a plant and hosts the most complex and vital biochemical process, photosynthesis. The typical shape is either round or disk-shaped, but the shape of the chloroplast can vary. Of the known yellow foliar soybean mutants, some seem to display defects in chloroplast size and shape. Chloroplasts in the *y11* mutant are around 6–8 μm in comparison to around 15 μm in a wild-type plant (Crang and Noble, 1974). The other mutant *cd1* doesn’t show reduction in overall size; however, the chloroplasts are irregularly oval-shaped (Zhang et al., 2011). For the *psbP* mutant, proplastids were round and pyriformed with dense stroma and did not develop into normal chloroplast (Sandhu et al., 2016). The *tic110* mutant displayed thinner and fewer chloroplasts with underdeveloped grana (Sandhu et al., 2016).

Thylakoids, the membranous structures present in the chloroplast, contain the pigments used in photosynthesis. In normal green soybean leaves, grana that are made of stacked thylakoids, contain anywhere from a few thylakoids to 10 or more. Grana are interconnected to one another via lamellae. Studies on different yellow foliar mutants revealed that the thylakoids and grana are commonly affected. Defects in two ATP dependent metelloproteases FtsH5 and FtsH2 that were shown to be involved in thylakoid membrane biogenesis in Arabidopsis resulted in variegated foliage mutants, *var1* and *var2*, respectively (Sakamoto et al., 2002; Aluru et al., 2006). The lipid monogalactosyl diacylglycerol (MGD) and phosphatidyl glycerol phosphate (PGP) are two of the components of the thylakoid membranes that are important for their structural and functional integrity (Maréchal et al., 1997; Jarvis et al., 2000). Syntheses of MGD and PGP are catalyzed by MGD synthase and PGP synthase, respectively (**Figure 2**). Mutations in genes coding for these enzymes caused yellow–green mutants in Arabidopsis (Maréchal et al., 1997; Jarvis et al., 2000). Two rice yellow–green mutants, *yl1* and *ys83*, that displayed problems in chloroplast development, were the results of mutation in a gene involved in biogenesis of chloroplast ATP synthase (cpATPase) (Chen et al., 2016; Ma et al., 2017) (**Figure 2**). Some proteins involved in signal transduction (UMP kinase), processing and stability of RNA (3-β-hydroxysterioid dehydrogenase/isomerase) and proteolysis (plastidic caseinolytic protease P6) have also been associated with chloroplast biogenesis and function in plants (Labesse et al., 2002; Dong et al., 2013; Shi et al., 2015; Zhu et al., 2016).

Some soybean mutants have less dense grana where only a few thylakoids are capable of attaching. For instance, in the *y3, y9, y11, cd1* and *psbP* mutants, the thylakoids remain unstacked to form single stack grana (Wolf, 1963; Keck and Dilley, 1970; Crang and Noble, 1974; Palmer et al., 1979; Eskins et al., 1983; Shoemaker et al., 1985; Jiang et al., 1997; Zhang et al., 2011; Sandhu et al., 2016). Probably, when the plant is lacking certain functional proteins it leads to disrupted thylakoid stacking. Different yellow foliar mutants show subtle differences in thylakoid stacking and chloroplast development.

### Defective Chloroplast Import Proteins

The Tic and Toc complexes are essential for proper chloroplast biogenesis as they are involved in the importing proteins from the cytosol (Bauer et al., 2000; Chen et al., 2002; Chou et al., 2003; Kovacheva et al., 2005; Kovacs-Bogdan et al., 2011). Defects in proteins involved in proper assembly of Tic and Toc complexes are known to be crucial for foliar pigmentation. In Arabidopsis the yellow foliar mutants, *y2, y3, y4*, and *y19* that are incapable of producing large number of chloroplasts are *tic20* mutants that contain decreased amounts of *Tic20* mRNA (Chen et al., 2002). The thylakoid membranes were reduced, fewer thylakoids were capable of stacking into grana, and the sizes of the plastids were also reduced. The *tic20* mutants also showed growth defects and were incapable of growing to their normal size (Kovacs-Bogdan et al., 2011). The reduction in Tic20 complexes also affected the import of three essential proteins for chloroplast: light-harvesting complex protein (LHCP), small subunit of ribulose-1,5-biphosphate carboxylase/oxygenase or RUBISCO (SSU), and α-subunit of the E1 subunit of pyruvate dehydrogenase (PDHα) (Chen et al., 2002). The total amounts of these proteins present within the chloroplasts were drastically reduced. Researchers argue that the reduction of the Tic20 complex is the main cause for the yellow phenotype and disruptions within the chloroplast (Chen et al., 2002; Kovacs-Bogdan et al., 2011) (**Figure 2**).

Independent knock out mutants of Tic40, Tic110, and Hsp90 complex genes in Arabidopsis were also explored for any developmental abnormalities (Kovacheva et al., 2005). The *tic40* mutants developed the most severe chlorotic phenotype out of the three; in fact, the mutant plants had about 1/3 chlorophyll content as compared to the wild-types (Chou et al., 2003; Kovacheva et al., 2005) (**Figure 2**). Mesophyll cells of the plants lacking the Tic40 complex contained reduced number and smaller (45% smaller) chloroplasts, thylakoids were spherical and appeared swollen, reduced thylakoid membrane networks and a very few grana stacks present. The *tic110* mutants are one of the few mutants that are lethal (Kovacheva et al., 2005). In addition, the mutants also showed retarded embryo development (delayed) compared to normal plants while very few embryos were capable of producing chloroplasts. Therefore, Tic110 is essential for embryo development and chloroplast biogenesis (Kovacheva et al., 2005; Kovacs-Bogdan et al., 2011). Phenotypically, the *tic110* mutants were shorter and contained reduced chlorophyll levels with fewer thylakoids which resulted in yellowish leaves (Kovacs-Bogdan et al., 2011) (**Figure 2**). The *hsp90* mutants did not depict severe phenotype but contained reduced chlorophyll and poorly developed chloroplasts (Kovacheva et al., 2005) (**Figure 2**). The protein importation capabilities of each complex were tested using 50S ribosomal subunits (L11) and RUBISCO proteins (SSU). The *tic40* and *hsp90* mutants had reduced importation of both proteins while *tic110* presented subtle decreases (Kovacheva et al., 2005). In addition, *tic40* also showed reduced importation of RuBP carboxylase, chlorophyll *a*/*b* binding protein (CAB), 33 kDa protein of oxygen-evolving complex (OE33) and POR (Chou et al., 2003). Hsp93 is known to interact with the import complex and may be critical of protein import (Kovacheva et al., 2005).

Toc complex, primarily made up of two GTP-regulated receptor proteins Toc34 and Toc159 along with a β-barrel membrane channel protein Toc75, recognizes and initiates the import of nuclear encoded proteins into the chloroplasts (Richardson et al., 2014). The *ppi2* mutant which lacked the *AtToc159* gene, developed a yellowish, pale phenotype with thylakoids that lacked membranes and starch granules (Bauer et al., 2000) (**Figure 2**). Protein import was also impacted by the lack of Toc159; two different proteins were employed to illustrate this effect. The *toc159* mutant displayed reduction in importation of chlorophyll *a*/*b* binding protein and both the small and large subunits of RUBISCO (Bauer et al., 2000). The proplastids were unable to develop into mature chloroplasts.

As mentioned above, most of the research on Tic and Toc complexes has been done in Arabidopsis. It is likely that some of the yellow foliar mutants identified in soybean may be defective for Tic and Toc proteins. Recently, in a soybean viable-yellow mutant (T380), a single base deletion was observed in the *Tic110* gene, causing a frameshift mutation that resulted in premature termination of encoded protein (Sandhu et al., 2016) (**Figure 2**). Future work on isolation of different soybean genes responsible for various yellow foliar mutants may shed light on the roles of different Tic and Toc proteins in soybean.

### Defects in the Electron Transport System

The components of electron transport system are directly involved in photosynthesis process in plants. Some mutants with defects in electron carrier proteins show yellow phenotypes. Electron acceptors are critical of energy generation process in an electron transport chain. A yellow–green mutant in rice, *501ys*, was the result of missense mutation in the *FdC2* gene that encodes ferredoxin-like protein (Li et al., 2015) (**Figure 2**). Ferredoxins are iron–sulfur proteins that are integral part of electron transport system in various metabolic pathways in a wide variety of organisms (Fukuyama, 2004). During photosynthesis, synthesis of NADPH via the electron transport system is an important step in plants. The knockout mutant of NAD kinase (*nadk2*) that catalyzes the *de novo* synthesis of NADP from NAD and ATP displayed reduced growth and pale yellow color in Arabidopsis (Chai et al., 2005) (**Figure 2**).

In soybean, extremely low F_v_/F_m_ values in *psbP* indicated complete lack of electron transport activity (Sandhu et al., 2016). Similarly, in *y11* mutant, the electron transport chain was shown to be defective (Keck et al., 1970). During photosynthesis, an electron acceptor, plastoquinone is reduced to plastoquinol and helps to transport the protons to the lumen of the thylakoids (Keck et al., 1970). The turnover rate for the oxidation of each plastoquinone molecule is drastically increased; which results in protons moving into the lumen faster (Keck et al., 1970). In addition, in the *y11* mutant the plastoquinone pool is also enlarged. By combining these two factors the *y11* yellow foliar mutant gene is capable of moving electrons faster than the wild-type soybean plant (Keck et al., 1970). The phenotype of such an increase results in light green leaves, reduced proteins, lower carotenoid values, decrease chlorophyll content, and altered thylakoid arrangements (Keck et al., 1970). Likewise, the defect in a chloroplast terminal oxidase that contains the ability to transfer electrons from the plastoquinol pool to oxygen resulted in green and white/yellow sectors in Arabidopsis leaves (Aluru et al., 2006).

### Temperature and Light Sensitive Yellow Alleles

The mutable gene *Y18-m* in soybean is considered unstable as it displays variegated yellow sectors on the leaves (Peterson and Weber, 1969; Palmer et al., 1979; Cheng, 1993; Kato and Palmer, 2004). The transition from *Y18-m* to *y18 or Y18* is regulated by temperature (Palmer et al., 1979). This sensitivity to temperature caused a germinal mutation to occur within the reproductive cells of the plant (Cheng, 1993; Kato and Palmer, 2004). The heterozygous *Y18-m y18* plants resulted into three different forms of gametes: *Y18-m, Y18*, and *y18* in various ratios (Peterson and Weber, 1969; Palmer et al., 1979; Cheng, 1993). When the allele was mutated it is the offspring, not the parents, which were directly impacted by this mutated allele and displayed either a normal or yellow phenotype. The phenotype of the offspring was completely dependent on the plant growth temperature. If the yellow seedlings were grown at 29°C the mutant gene had a drastic impact on chloroplast content and development, and the grana and lamellar systems were disrupted (Palmer et al., 1979; Shoemaker et al., 1985). The chlorophyll content was reduced by half, but the ratio of chlorophyll *a*/*b* remained unchanged. If the yellow seedlings were grown at 19°C the thylakoid lumen fluctuated in size and there was an increase in the number of osmiophilic bodies present (Palmer et al., 1979). Therefore, the severity and hindrances produced within the chloroplast in yellow plants was completely dependent on what temperature the plants were grown at. Similarly, the chlorophyll deficient mutant, *cde1*, in rice was shown to be thermosensitive (Liu et al., 2007). The mutant showed normal phenotype at 23°C or lower, but at 26°C or higher the plant exhibited a yellow–green foliar phenotype. A glutamyl-tRNA synthase (*OsGluRS*) gene was shown to be responsible for this phenotype (Liu et al., 2007) (**Figure 2**). Certain yellow mutants showed the direct impact of illuminance on a soybean plant. A rice mutant *light-induced yellow leaf1-1* (*lyl1-1*) was due to a mutation in geranylgeranyl reductase that is involved in reduction of Chl-geranylgeranylated (Chl_GG_) and geranylgeranyl pyrophosphate (GGPP) to Chl-phytol (Chl_Phy_) and phytyl pyrophosphate (PPP), resulting into defect in chlorophyll synthesis (Zhou Y. et al., 2013) (**Figure 2**). As *LYL1* can be induced by light, it is believed to be critical in response to high light in rice (Zhou Y. et al., 2013). When exposed to high amounts of sunlight, the soybean mutants *y11* and *y18-m* displayed absence of thylakoid stacking and lack of ribosomes in the stroma (Koller and Dilley, 1974; Palmer et al., 1979). In fact, the total amount of chlorophyll was reduced up to 10-fold with excessive illuminance (Koller and Dilley, 1974). Similar observations were made for the *tic110* mutant where there were distinct phenotypic differences between the wild-type and the mutant at later growth stages under field conditions; however, phenotypes were indistinguishable in a greenhouse (Sandhu et al., 2016).

### Other Proteins Involved in Photosynthesis

In several of the mutants, defects in some essential proteins may negatively impact photosynthesis. RUBISCO regulates photosynthesis by fixing carbon dioxide into energy-rich glucose molecules, which is found to be directly proportional to the photosynthetic rates of the plant (Johnson, 2016). In the fully expanded leaves of mutants *y3* and *y11*, overall RUBISCO content was significantly reduced in comparison to the wild-type (Jiang et al., 1997). Other proteins that are affected in the *y3* and *y11* soybean mutants include CAB proteins (light harvesting chlorophyll *a/b*-binding proteins of PS II), RUBISCO activase, beta subunits of the chloroplast ATP synthase and cytochrome *f*. In *y11*, the RUBISCO activase, beta subunits and cytochrome *f* proteins decreased drastically throughout the leaf development; while the CAB proteins progressively increased over time (Jiang et al., 1997). The *y3* mutant displayed a reduction in all four proteins listed above throughout the leaf development. The decline in these specific proteins negatively impacted the plant’s ability to undergo photosynthesis (Jiang et al., 1997).

The methylerythritol phosphate (MEP) pathway is involved in isoprenoid biosynthesis and is closely linked to photosynthesis as it uses its precursors and electrons from photosynthesis (Seemann et al., 2006). The *methylerythritol 2,4-cyclodiphosphate synthase* (*IspF*) gene was shown to be responsible for the yellow–green phenotype in rice mutant *505ys* (Huang et al., 2018) (**Figure 2**). IspF catalyzes the conversion of 4-diphosphocytidyl-2-C-methyl-D-erythritol-2-phosphate (CDP-MEP) into 2-C-methyl-D-erythritol-2,4-cyclodiphosphate (ME-cPP) in the MEP pathway for isoprenoid biosynthesis (Herz et al., 2000).

### Can Yellow Foliage Mutants Have Positive Effect on Soybean Productivity?

Studies on light absorbance and utilization in soybean revealed that excessive chloroplast present in the top layer of leaves reduces the amount of sunlight that reaches the lower leaves (Pettigrew et al., 1989). The measurement of canopy photosynthetic CO_2_-exchange rates (CER) determines how much photosynthetic proton flux density (PPFD) will pass through the leaves. If more PPFD is capable of passing through the chlorophyll-deficient leaves and reaches the lower leaves it will result in a higher overall CER value. The *y9* and *y11* mutants had up to 20 and 38% higher CER daily, respectively, compared to wild-types (Pettigrew et al., 1989). In a recent study performed in field conditions, decreased chlorophyll contents in *y9* and *y11* provided transient benefits by increasing leaf-level photosynthesis earlier in the growing season (Slattery et al., 2017). However, no improvement was seen in yield of mutant’s *y9* and *y11* in comparison to wild-type soybean, though these years were severely affected by drought (Slattery et al., 2017). Authors concluded that soybean plant overinvests in chlorophyll, as 50% reduction in chlorophyll did not significantly reduce yield and biomass accumulation; which means that relocation of nitrogen from pigment-protein complexes to other molecules involved in photosynthesis may be beneficial for plant productivity (Slattery et al., 2017). These conclusions are based on two mutants and single location. Multi-location trials including additional yellow mutants are necessary, which will provide definite conclusive evidence on the performance and yields of yellow mutants. Since, different mutants are operating in different pathways, a particular type of yellow mutant may be better than others in distributing light more proportionately in different leaf layers. Future studies targeting chlorophyll reduction and directing important resources toward increasing biochemical photosynthetic capacity are warranted to increase soybean productivity.

### Insights From Model Plants and Perspective Applications in Soybean

Insights from model systems can be invaluable in understanding mechanisms and pathways leading to yellow phenotypes in soybean. At least, 28 nuclearly inherited yellow foliar mutants have been identified in soybean, of which only four corresponding genes have been cloned. These include genes encoding for a magnesium-chelatase subunits ChlI1a and ChlI1b (*Y11* and *CD-5*), translocon in the inner membrane of chloroplast (*Tic110*) and an extrinsic protein of PSII (*PsbP*) (Reed et al., 2014; Campbell et al., 2015; Sandhu et al., 2016) (**Figure 2**). Molecular and genetic research in model plants such as Arabidopsis and rice resulted in isolation and characterization of a number of genes associated with yellow phenotype. These include genes involved in chlorophyll biosynthesis, thylakoid biogenesis, lipid synthesis, RNA processing, proteolysis, transport proteins, components of electron transport chain, assembly and biogenesis of photosystems and signal transduction. Some of the characterized genes are associated with pathways seemingly distant from photosynthetic process. For example, the mutant *IspF* gene that is involved in isoprenoid synthesis through MEP pathway resulted in yellow phenotype in rice (Huang et al., 2018). Characterization of this gene established a link between photosynthesis and MEP pathway showing that photosynthesis is the source of electrons needed for the MEP pathway (Seemann et al., 2006) (**Figure 2**). The information generated for the isolated yellow foliar genes in plants clearly revealed that the structures and functions of various genes are conserved in different plant species. For instance, the rice yellow foliar mutant *chlorina-9* is defective in magnesium chelatase subunit ChlI, homologs of which are shown to be responsible for the yellow green phenotype in two soybean mutants *y11* and *CD-5* (Zhang et al., 2006; Campbell et al., 2015) (**Figure 2**). Similarly, *tic110* mutants display yellow foliar phenotypes in Arabidopsis and soybean (Sandhu et al., 2016). Likewise, the *OsFdC2* gene in rice is also a functional ortholog of *AtFdC2* (Li et al., 2015; Zhao et al., 2015) (**Figure 2**). As there are a number of yellow mutants identified in soybean, the homology based cloning approach will be highly effective in isolating and characterizing corresponding genes from soybean. The ease of transformation, availability of genetic resources, the body of information and availability of fully annotated genome makes Arabidopsis an ideal system to perform mechanistic studies on these genes. Lessons learnt from model plants can help in providing insights into the evolution of genetic networks involved in photosynthesis, a complex metabolic process vital for existence of plants. Characterization of yellow foliar mutants will provide effective means of understanding the modes of actions of proteins in metabolic pathways associated with foliage color in soybean. This, in turn, could explain the metabolic profiles of the sugar synthesis process in plants and facilitate in elaborating the underlying physiological and biochemical mechanisms. With the present attention and effort to understand the function of all genes in crop plants, the functional characterization of more genes involved in the yellow foliar phenotype may provide important tools to decipher connections between different metabolic pathways. As this has already been recognized that future gain in crop productivity will involve increasing efficiency of photosynthesis, the generation of new knowledge about the photosynthesis process will be the key to improved productivity of soybean and other crops in future.

## Conclusion

Soybean yellow foliar mutants present an interesting opportunity to understand the complex photosynthesis process due to their compromised ability to undergo photosynthesis. Different mutants represent wide variety of defects in different proteins involved in transport, assembly of photosystems, pigment biosynthesis, chloroplast development, electron transport chain, and catalysis of important reactions involved in photosynthesis. Phenotypically, all mutations affect the concentration of chlorophylls and/or other pigments in leaves. The ratios between chlorophyll *a* and the other pigments have also been altered in some of the yellow mutants. Certain mutants display interruption in thylakoid stacking in chloroplast or isolate grana from each other due to problems with lamellae, thereby negatively affecting the ability to harvest light. Any problem with this process of channeling absorbed light energy to generate chemical reducing power can lead to excessive energy that can cause oxidative damage to the thylakoids resulting in photoinhibition. Underdeveloped chloroplasts and reduced photochemical efficiency in some mutants may be the cause of yellowing due to the light stress. Mutations in Tic and Toc complexes, that play important roles in translocating proteins into the chloroplast, are known to cause problems in chloroplast development in plants. Future work on soybean yellow foliar mutants may uncover the importance of chloroplast proteins in soybean photosynthetic pathway. Although, there are many negative consequences of yellow foliage mutations, some recent studies suggest that some of these mutants can be utilized to increase canopy photosynthetic CO_2_-exchange rates under field conditions. This may result in more efficient utilization of the light energy and possibly result in increased net productivity.

Future studies geared toward using knowledge gained from model plants may help in targeting different mechanisms and pathways to identify candidate genes associated with yellow foliar phenotype in soybean. Mapping, isolation, and functional characterization of genes involved in foliar pigmentation may enhance our understanding of the photosynthetic mechanism. It may help us understand how decreased chlorophyll content can affect other proteins and processes. Perhaps new advances in the knowledge about the photosynthesis process may help in developing soybean germplasm to maximize light energy capture efficiency; which can directly translate into increased productivity.

## Author Contributions

DS and ZC wrote the initial draft of the manuscript. TA, KR, and VM edited the manuscript. The final draft of the manuscript was approved by all authors.

## Conflict of Interest Statement

The authors declare that the research was conducted in the absence of any commercial or financial relationships that could be construed as a potential conflict of interest.

## Footnotes

1 www.nass.usda.gov
